# An optimized GATK4 pipeline for *Plasmodium falciparum* whole genome sequencing variant calling and analysis

**DOI:** 10.1186/s12936-023-04632-0

**Published:** 2023-07-07

**Authors:** Karamoko Niaré, Bryan Greenhouse, Jeffrey A. Bailey

**Affiliations:** 1 Department of Pathology and Laboratory Medicine, Brown University, Providence, RI USA; 2 Center for Computational Molecular Biology, Brown University, Providence, RI USA; 3 EPPIcenter Program, Division of HIV, Infectious Diseases, and Global Medicine, Department of Medicine, University of California San Francisco, San Francisco, CA USA

## Abstract

**Background:**

Accurate variant calls from whole genome sequencing (WGS) of *Plasmodium falciparum* infections are crucial in malaria population genomics. Here a falciparum variant calling pipeline based on GATK version 4 (GATK4) was optimized and applied to 6626 public Illumina WGS samples.

**Methods:**

Control WGS and accurate PacBio assemblies of 10 laboratory strains were leveraged to optimize parameters that control the heterozygosity, local assembly region size, ploidy, mapping and base quality in both GATK HaplotypeCaller and GenotypeGVCFs. From these controls, a high-quality training dataset was generated to recalibrate the raw variant data.

**Results:**

On current high-quality samples (read length = 250 bp, insert size = 405–524 bp), the optimized pipeline shows improved sensitivity (86.6 ± 1.7% for SNPs and 82.2 ± 5.9% for indels) compared to the default GATK4 pipeline (77.7 ± 1.3% for SNPs; and 73.1 ± 5.1% for indels, adjusted P < 0.001) and previous variant calling with GATK version 3 (GATK3, 70.3 ± 3.0% for SNPs and 59.7 ± 5.8% for indels, adjusted P < 0.001). Its sensitivity on simulated mixed infection samples (80.8 ± 6.1% for SNPs and 78.3 ± 5.1% for indels) was again improved relative to default GATK4 (68.8 ± 6.0% for SNPs and 38.9 ± 0.7% for indels, adjusted, adjusted P < 0.001). Precision was high and comparable across all pipelines on each type of data tested. The resulting combination of high-quality SNPs and indels increases the resolution of local population population structure detection in sub-Saharan Africa. Finally, increasing ploidy improves the detection of drug resistance mutations and estimation of complexity of infection.

**Conclusions:**

Overall, this study provides an optimized *falciparum* GATK4 pipeline resource for variant calling which should help improve genomic studies of malaria.

**Supplementary Information:**

The online version contains supplementary material available at 10.1186/s12936-023-04632-0.

## Background

Malaria’s toll on human health remains unacceptably high with 627,000 deaths reported worldwide in 2020 [1]. Despite substantial progress in control made during the past two decades, decreases in mortality have slowed or reversed [1]. The vast majority of estimated deaths continue to occur in sub-Saharan Africa in children aged below 5 years due to the most virulent species, *Plasmodium falciparum*. Control efforts are challenged by the lack of highly effective vaccines, drug and insecticide resistance, failure of rapid diagnostic tests due to *hrp2* and *hrp3* deletions, and the impact of the COVID-19 pandemic [1–4]. Further progress necessitates improved tools for furthering control and for the monitoring of progress towards eventual elimination.

Whole genome sequencing (WGS) represents a key tool for genomic epidemiology and surveillance of parasite populations that circulate in malaria endemic regions. Significant advances have been made in malaria research since the first WGS data of *P. falciparum* was released in 2002 to provide a reference genome [5]. Today, laboratory and large clinical WGS datasets of parasites representing malaria infections are generated predominantly by Illumina next-generation sequencing [6]. Studies based on WGS have been instrumental in defining and understanding drug resistance, including the discovery of *kelch 13 (k13)* mutations as the molecular markers of artemisinin resistance as well as providing a detailed description of the parasite population structure within and across continents [6–8].

A crucial step in WGS data analysis is calling variants by mapping reads onto a reference genome and looking for consistent differences representing single nucleotide polymorphisms (SNPs), insertions–deletions (indels) and structural variations. First*,* the genome of *P. falciparum* is AT-rich with large regions of low complexity and duplicated sequence, such as the subtelomeric regions which contain highly-related gene families. This presents a general challenge for mapping reads accurately to the reference genome. The *P. falciparum* genome is extremely repetitive in many (coding and non-coding) regions, with high frequencies of AT-rich tandem repeats that create challenges for accurate calling of variants [9, 10]. Indels are predominantly associated with short AT-rich sequence repeats and are challenging to accurately identify with short read sequencing. Consequently, SNPs, which are relatively less difficult to identify compared to indels, have been the preferred variant type for *P. falciparum* population genetics studies. Second, sequence data from natural malaria infections present an additional challenge, often containing an unknown number of clones (complexity of infection, COI) at unknown relative abundances [5, 11, 12].

Despite the fact that *P. falciparum* is entirely haploid in the human host during its lifecycle, the performance of the variant calling is affected by the high COI of clinical samples collected from patients in areas of high malaria transmission. The number of clones can sometimes reach double digits, and given their numbers and relative frequency are variable and unobserved, standard variant calling pipelines based on ploidy do not properly apply. Moreover, given the proportion of an individual clone within an infection can be extremely low, the detection of its presence and variants it may contain can be computationally challenging.

The genomic analysis toolkit (GATK) [13, 14] is a well-validated tool that has been traditionally used to call variants (SNPs and short indels) in malaria WGS data. However, this tool was initially developed for diploid human and other mammalian genomes and has not been extensively optimized for microorganisms, particularly challenging ones such as *P. falciparum*. One of the most important features of GATK is the variant recalibration step which consists of using a training dataset to score and filter out low quality variants that are more likely false than true from the raw variant data in variant call format files (VCFs). However, developing a Mendelian error-free (or error-depleted) training dataset based on sequence reads generated in the wet laboratory is currently a challenge in malaria. Additionally, GATK was designed for diploid organisms, where there are equal proportions of each chromosome present in the sample; in natural malaria infections with COI > 1 it is more difficult to distinguish the few copies of actual variants in minor clones from sequencing errors. This issue could be partially overcome by partitioning the ploidy until each expected variant present in the sample gets enough probability to be called.

Here the flexibility of GATK4 was leveraged to optimize SNP and indel calling for improved *P*. *falciparum* WGS data analysis. A custom variant training dataset in silico was generated based on accurate PacBio assemblies of laboratory strains as templates to filter out low quality variants. Prior variant rates and multiple parameters that control the read and mapping qualities were adjusted at various stages of the pipeline. A reference true callset was also made with select known laboratory strains to evaluate the performance of the pipeline. Finally, the pipeline was applied to available public WGS data, including the Malariagen Pf6 dataset, demonstrating improvements in population genomic analyses. Since indels have been challenging to properly call and rarely incorporated into these analyses, the study focuses on them and demonstrates their utility in increasing the resolution of population structures when combined with SNPs.

## Methods

### Reference callset (“gold standard”) generation

Publicly available accurate PacBio assemblies of 10 laboratory strains of *P. falciparum*, including 7G8, Dd2, GA01, GB4, GN01, HB3, IT, KH01, KH02 and SN01 [15, 16] in fasta format were aligned onto the *P. falciparum* 3D7 reference genome (Version 3) with **minimap2** version 2.17. Mapped single chromosome-long reads were used to retain all mismatches as variants using optimized **bcftools** (version 1.13) **mpileup** and **bcftools call** commands. Variant calling was validated by visualizing VCFs and BAMs together in **IGV** (version 2.4.17) to confirm the concordance between variants and mismatches. Subtelomeric and internal hypervariable regions were removed as these regions undergo frequent exchange between chromosomes such that orthology is not maintained.

### New in silico training dataset generation from PacBio assemblies

The same 10 PacBio assemblies were harnessed as templates to computationally generate a positive training dataset for GATK. **Biopython** scripts were used to make 2 kb fragments of each genome after every 20 nucleotide positions and fragments were saved as fasta files. This strategy produces overlapping 2 kb reads with 100× coverage from which fastq files were created by setting the quality of bases to highest quality (“~” in ASCII format) using **bbmap**. These reads were mapped onto the 3D7 reference genome using **minimap2** (version 2.17). BAM files were sorted and duplicate reads marked using tools Picard **SortSam** and GATK **MarkDuplicatesSpark** prior to variant calling, respectively. The **HaplotypeCaller** package of **GATK** version 4.1.6.0 was used to call potential variants and create genome VCFs (gVCFs). The 10 gVCFs were combined using **GenomicsDBImport** (GATK4’s tool) before running a joint genotyping with **GenotypeGVCFs** (GATK4’s tool) within the core genome. The final VCF was compared to the reference callset for each laboratory strain using **RTG Tools** to verify the quality of the training SNPs and indels. This tool was employed for all subsequent VCF comparisons, in which the accurate reference callsets were used as baselines along with the following arguments -**decompose** and -**squash-ploid**y that normalize complex variants into smaller constituents and ignore zygosity differences between VCFs that are compared, respectively. The output data of RTG Tools analysis for different pipelines tested on multiple laboratory strains (single and mixed infections) were compared using Wilcoxon test with the **rstatix** R package.

### Developing an optimized variant calling pipeline

The application of an optimized pipeline to Illumina sequencing is illustrated in Additional file 1: Figure S5. Code for the pipeline is available (https://github.com/Karaniare/Optimized_GATK4_pipeline). Illumina reads of laboratory stains and Pf6 samples were downloaded from SRA using **sratoolkit** (version 2.8.2-1). Trimmed paired reads were competitively aligned to the *P. falciparum* 3D7 and human (hg38) reference genomes with **bwa** (version 0.7.15). Reads mapping specifically onto the 3D7 genome were selected before cleaning the bam files using **CleanSam**. Clean BAM files were sorted and processed for duplicate marking using **SortSam** and **MarkDuplicatesSpark,** respectively. Mixed infection samples were simulated after combining 95%, 90%, 85%, 80%, 75% and 50% mapped reads of the KH01 strain with 5%, 10%, 15%, 20%, 25% and 50% mapped reads of IT strain to make final BAMs with 100× coverage. To optimize **HaplotypeCaller** (**GATK**4) for initial variant calling from *P. falciparum* WGS data with higher sensitivity, multiple parameters were adjusted as follows: -**heterozygosity** (prior SNP rate) 0.0029, -**indel-heterozygosity** (prior indel rate) 0.0017, -**min-assembly-region-size** 100, **min-base-quality-score** (minimum base quality required to consider a base for variant calling) 5 and **-base-quality-score-threshold** 12. Heterozygosity values were chosen based on SNP and indel rates calculated from the reference callsets of the 10 laboratory strains. Base quality score filtering thresholds were made less stringent to allow the algorithm to process higher amounts of reads. The mapping quality filter (**-DF** MappingQualityReadFilter) was also disabled for the same reason. Optimized HaplotypeCaller was run on each sample independently to initially detect potential variants that are stored in gVCFs. For joint genotyping option, gVCFs were first combined into a genomic database using **GenomicsDBImport**. Per-chromosome genotyping of the gVCFs was performed using the **GenotypeGVCFs** command in which **-stand-call-conf** (minimum phred-scaled confidence threshold at which variants should be called) was set to 30 to remove the majority of false positive variants that passed **HaplotypeCaller**’s filters. The -**genomicsdb-use-vcf-codec** argument in the joint genotyping script was enabled to allow for more space in info fields when annotation sizes exceed 32-bit while processing the genomic database. Since the genotyping step is computationally costly, **GenotypeGVCFs** was run in parallel **slurm** jobs on each chromosome partitioned into 200 kb segments. **GATK4**’s **GatherVcfs** was used to merge segments into single VCFs. When indels and SNPs are called at the same positions, they are recorded as multi-allelic and incorrectly treated as indels by GATK’s VCF filtering engine. To accurately filter low-quality (likely false) SNPs and indels independently, **bcftools norm** commands were used to split multi-allelic positions before applying **GATK4**’s **VariantRecalibrator** and **AppyVQSR**. The **VariantRecalibrator** analysis was performed based on **QD**, **DP**, **FS**, **SOR** and **MQ** using either the public cross data (downloaded from ftp://ngs.sanger.ac.uk/production/malaria/pf-crosses/1.0/) or the in silico positive training dataset made with the 10 laboratory strains. The -**lod-score-cutoff** (VQSLOD cutoff) in **AppyVQSR** was set to 0 for SNP and − 2.0 for indel (due to the mapping issue of the low complexity repeat regions) to annotate low quality variants. Low quality variants (VQSLOD < 0 for SNP and VQSLOD < − 2.0) were tagged with the annotation “LOW_VQSLOD” and can be used to remove them from the VCFs. The command **bcftools norm** was used to merge variants with the same positions into multi-allelic loci as multi-position VCFs are hard to handle in downstream analyses. The final VCFs were functionally annotated with **SnpEff** (version 5.0d). **Bcftools view** and **bcftools query** were used for all VCF subsetting and data extraction and the outputs were plotted with R Studio.

### Increased ploidy GATK4 analysis of drug resistance

The optimized pipeline was run on Pf6 data with -**ploidy** argument of **HaplotypeCaller** set at 6 (hexaploid mode) in comparison to 2 (diploid mode). Validated *k13* mutations associated with artemisinin resistance were analysed. The annotated VCF was subset at *k13* gene locus on chromosome 13 (1,724,600–1,727,877) using **bcftools view** and amino acid changes and positions were extracted with **SnpEff**’s **SnpSift.jar** package. In order to identify samples that carry these mutations, **GATK4**’s **VariantsToTable** was used to extract the **GT** information along with **REF** and **POS** into a table. The same approach was applied to analyse k13 mutations in MalariaGEN’s Pf6 GATK3 VCF which was downloaded from the publicly available repository ftp://ngs.sanger.ac.uk/production/malaria/pfcommunityproject/Pf6/Pf_6_vcf/. For mutated samples that were not found in the GATK3 VCF, BAMs were visualized in **IGV** to confirm the mutations were present across multiple reads.

### Complexity of infection analysis in field isolates

The command **bcftools view** was used to extract SNPs with minor allele frequencies > 1% from chromosome 13 in VCFs which included 6626 samples from West Africa, East Africa, Central Africa, Central West Africa, South-East Africa, South-East Asia West, South-East Asia East, South America, South Asia and Papua_New_Guinea. The command **bcftools + fill-tags** was used to add fractions of missing genotype annotations to the VCFs and **bcftools view** to select SNPs with less than 10% missingness rates. Custom scripts based on **vcftools** were used to create genotype tables from diploid and hexaploid VCFs in which reference homozygous, alternate homozygous and heterozygous calls were coded as 0, 2 and 1. Finally, the genotype tables were used to compute discrete COIs with the **REAL McCOIL** package [23] in **jupyter notebook.**

### Population structure analysis

To analyse the population structure, variants were pruned for linkage disequilibrium and samples for missingness (> 20%). SNPs and indels of chromosome 1 were tested individually and in combination to assess the impact of both types of variants combined on the population structure. Multiallelic sites in SNP or indel-specific VCF were split and optimized **PLINK** codes were used to compute the variance-standardized genetic relationship matrix between pairs of African samples. The variance-standardized genetic relationship matrix was used to conduct tSNE analysis at a perplexity of 30 and a learning rate of 200 using the **Rtsne** package in R. The tSNE dimensions were extracted and plotted with **ggplot2**.

## Results

### Optimization of the pipeline on monoclonal and simulated mixed infection samples

Towards optimizing GATK4 for *P. falciparum*, the creation of an improved training “truth set” for the pipeline was key. To filter raw VCFs with a high quality truth callset, which is difficult to obtain using wet laboratory methods, a robust in silico training variant dataset was generated from the PacBio assemblies (Additional file 1: Figure S1). First, the variant calling pipeline was run with default settings of GATK4’s HaplotypeCaller and GenotypeGVFs tools on high-quality public Illumina read data (Additional file 1: Table S1) of 10 laboratory strains (7G8, Dd2, GA01, GB4, GN01, HB3, IT, KH01, KH01 and SN01) for which there exist accurate PacBio assemblies [15, 16]. The raw VCFs were recalibrated with the published training dataset generated from 3D7 × HB3, HB3 × Dd2 and 7G8 × GB4 crosses in splenectomized chimpanzees [17–19] and used by the MalariaGEN Pf6 release [6, 9]. The results were compared to reference callsets generated from the accurate PacBio assemblies mapped onto the 3D7 reference (“Methods”, Additional file 1: Figure S2).

**Fig. 1:**
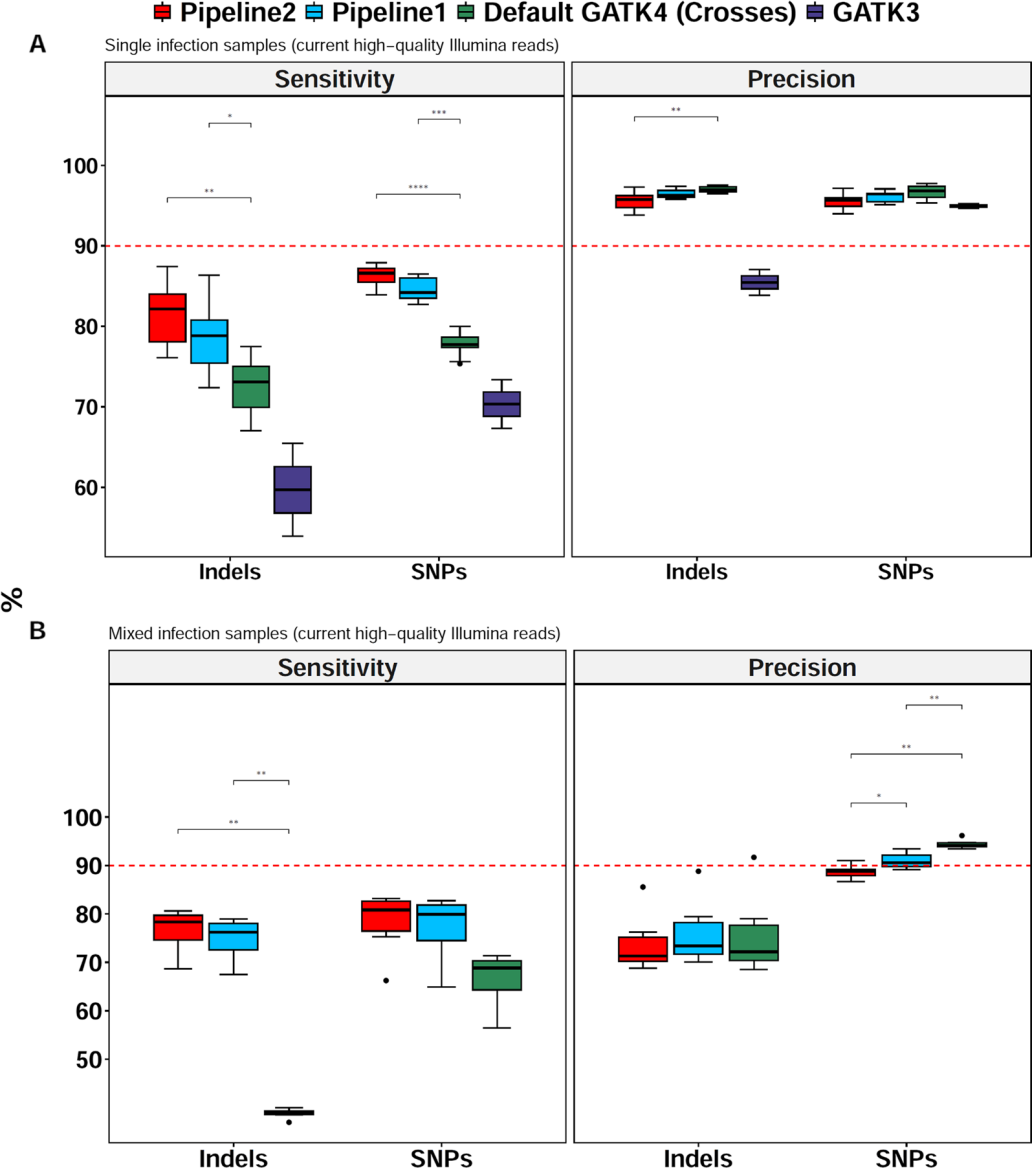
Performance of the optimized GATK4, default GATK4 and GATK3 pipelines. **A** Pipeline performance using current high-quality Illumina read data (read length = 250 bp; insert size = 405–524 bp) from single infection samples. Ten laboratory strains (7G8, Dd2, GA01, GB4,GN01, HB3, IT, KH01, KH02 and SN01) were included for all the pipelines except GATK3 as only two (GN01 and KH02) of these samples were found in the available GATK3 VCFs on the MalariaGEN website. **B** Pipeline performance on simulated high-quality mixed infections samples of IT + KH01 at 95:5, 90:10, 85:15, 80:20, 75:25, and 50:50 proportions (100× read depth). Only significant statistical differences obtained with the Wilcoxon test are shown (indicated by asterisks). Pipeline 1: GATK4 pipeline with default settings of HaplotypeCaller and GenotypeVCFs coupled with variant recalibration by the in silico training dataset. Pipeline2: fully optimized GATK4 pipeline with alternation of HaplotypeCaller and GenotypeGVFs parameters and variant recalibration (filtering) using the new in silico training dataset. Default GATK4 (crosses): Default GATK4 pipeline but recalibrated by the publicly available cross dataset. GATK3: same GATK3 pipeline used by MalariaGEN’s Pf6 release in which variants are recalibrated by the cross training dataset. Red dashed line represents 90% performance

Sensitivity of this default variant calling in the core genome were 77.7 ± 1.3% (median ± interquartile range) for SNPs and 73.1 ± 5.1% for indels (Fig. 1A). When the cross dataset was replaced with the PacBio-derived in silico training dataset (called pipeline 1), sensitivity was greatly improved (84.2 ± 2.5% for SNPs; and 78.8 ± 5.4% for indels). With GATK3 recalibrated with the cross training dataset, the sensitivity for both SNPs and indels was the lowest. As next step in GATK4 optimization, multiple parameters of HaplotypeCaller and GenotypeGVFs of pipeline 1 were altered to make pipeline 2, including adjusting expected SNP and indel rates (-heterozygosity and -indel-heterozygosity) and parameters that control base quality (-base-quality-score-threshold and -stand-call-conf), mapping (-mbq and -DF) and local assembly size (-min-assembly-region-size), aiming to further improve the sensitivity and reduce false calls. While default values of pipeline 1 are fairly robust, sensitivity increased significantly to 86.6 ± 1.7% for SNPs and 82.2 ± 5.9% for indels when modified parameters were used (“Methods”; Fig. 1A; adjusted P < 0.001, Wilcoxon test, Pipeline 2 vs. default GATK4 with cross training dataset for both SNPs and Indels). Despite the trade off between sensitivity and precision, the latter was very high (> 90%) for all GATK4 pipelines, including the new pipelines 1 and 2.

The GATK4 pipelines were finally tested on simulated mixed infection samples after combining high-quality reads of IT + KH01 at different proportions (95:5, 90:10, 85:15, 80:20, 75:25 and 50:50) to make 100× coverage. Sensitivity of the optimized pipelines was higher than that of the default GATK4 pipeline trained with the cross dataset for both SNPs and indels (adjusted P = 0.001, Wilcoxon test) (Fig. 1B). Interestingly, the most significant performance gains were observed with indels for which the sensitivity of pipeline 2 was 78.3 ± 5.1% versus 38.9 ± 0.7% for default GATK4 with cross training dataset (Fig. 1B, adjusted P < 0.001, Wilcoxon test). Here the precision was similar across all GATK4 pipelines but lower compared to single infection samples.

**Fig. 2:**
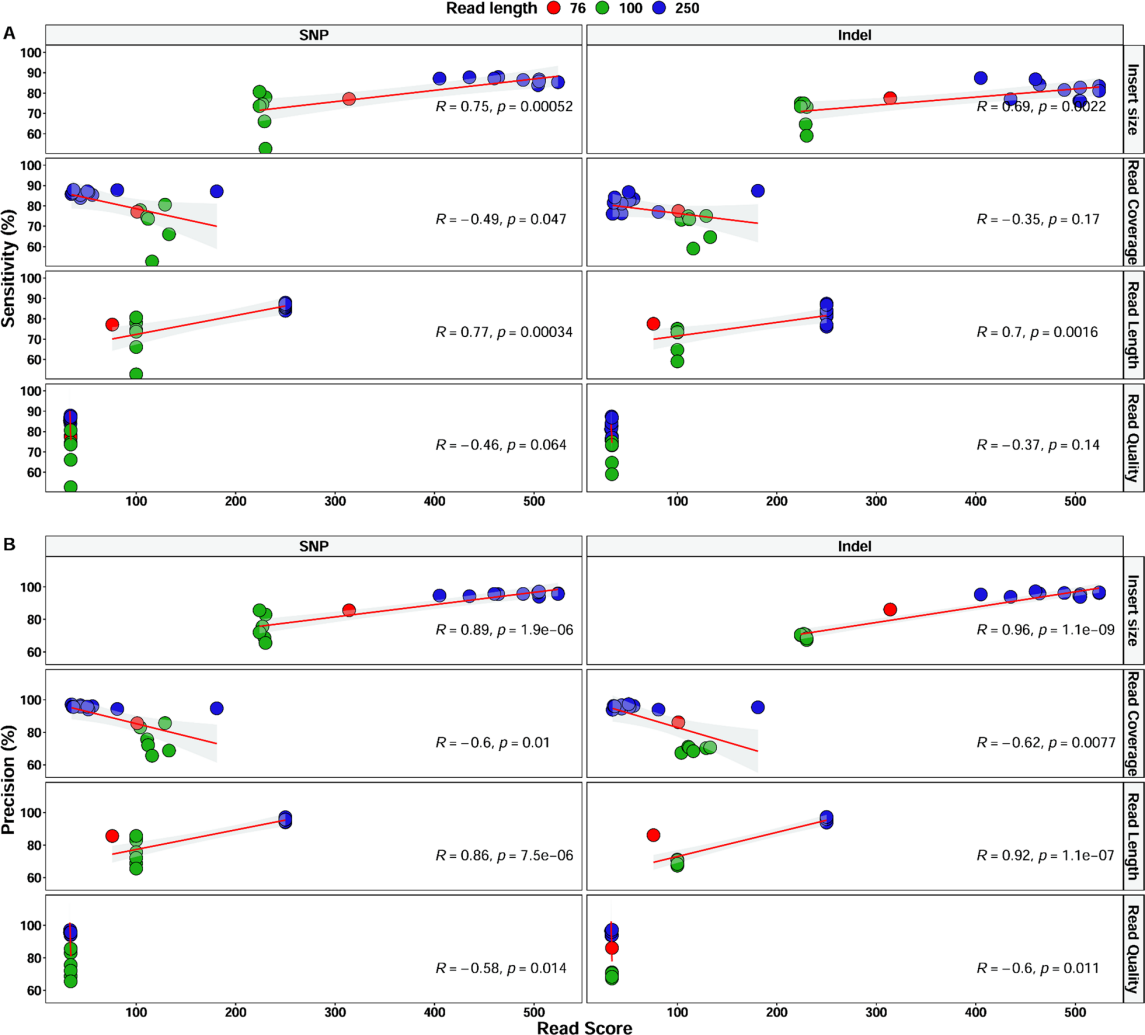
Correlation analysis between sequencing quality parameters and variant calling performance, sensitivity (**A**) and precision (**B**). Red (75 bp) and green (100 bp) represent samples with old shorter Illumina reads. Samples with current longer Illumina reads are coloured blue. Read score in the x-axis represents values for either insert size, read coverage, read length or read quality. Pearson correlation was used

To understand how sequencing quality affects the results of the pipelines, shorter (old) Illumina read samples (n = 7) from the Pf6 dataset were added and analysed for any potential effect of the read coverage, insert size, read length and read quality on variant calling performance (Additional file 1: Table S2). In spite of the great variation in the read coverage (median between 35 and 180×), neither the specificity nor the precision was correlated with this parameter (Fig. 2). Interestingly, a strong positive correlation was found between pipeline performance and insert size and read length (Fig. 2).

### Combination of SNPs and indels shows higher resolution of population structure

**Fig. 3:**
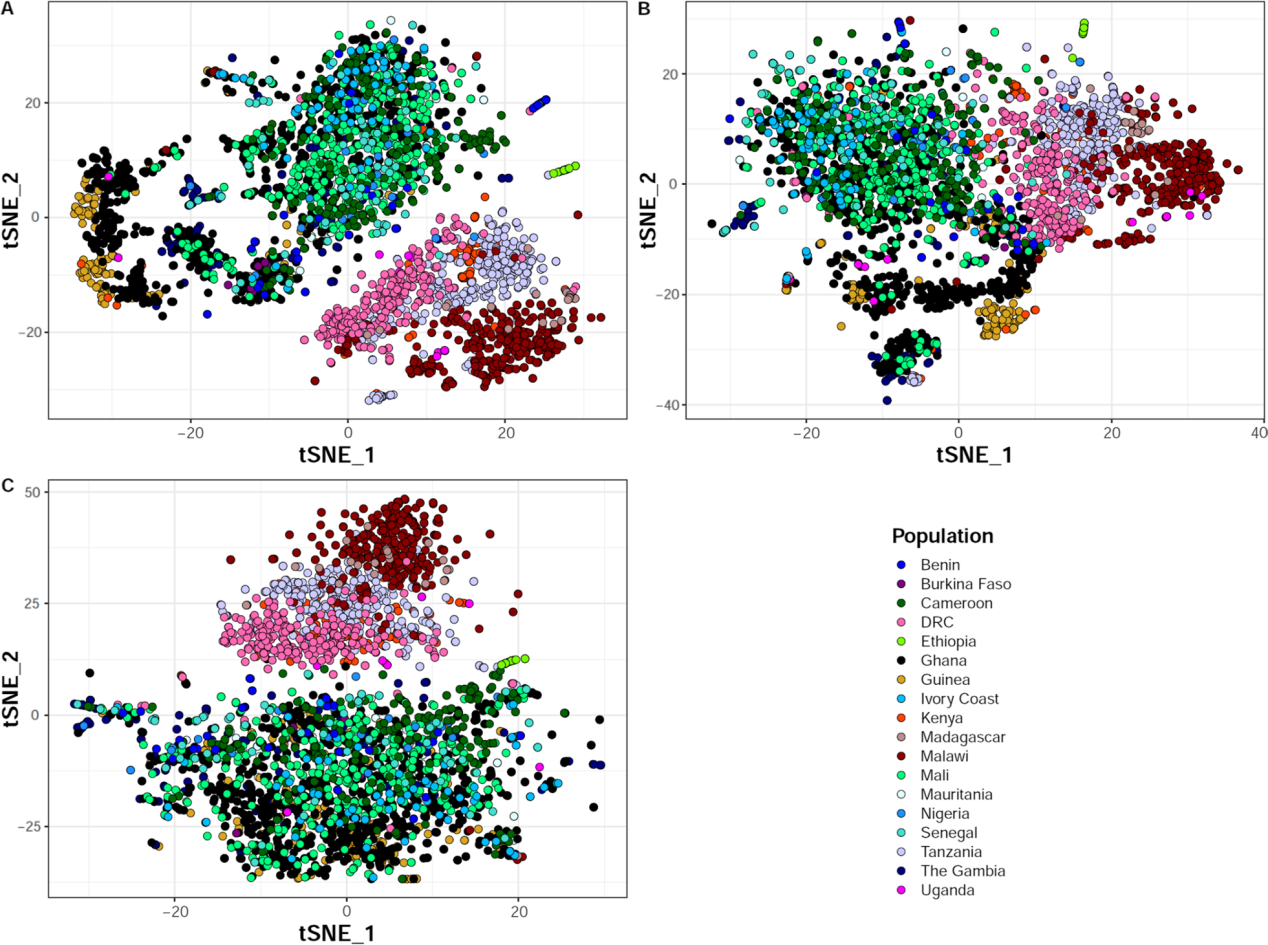
Local population structure in sub-Saharan Africa. t-distributed stochastic neighbour embedding (tSNE) was computed from the variance-standardized genetic relationship matrix generated using **A** SNPs and indels combined, **B** indels only and **C** SNPs only. Variant data (from chromosome 1) were pruned for linkage disequilibrium and only samples with less than 20% missing genotypes (n = 3008) were kept. DRC: Democratic Republic of Congo

Since the performance of indel calling was markedly improved with GATK4, the inclusion of indels was examined for improved resolution of global and local population structure using Malariagen’s Pf6 samples. After restricting the analysis to the core genome and filtering out low quality variants; the fully optimized pipeline detected more variants in total compared to GATK3. The majority (55%) of indel positions overlapped with each other and with SNP sites and formed complex multiallelic markers (2,341,377 SNPs, 1,381,687 indels and 1,686,041 multiallelic sites in 6626 samples for the pipeline; and 2,441,874 SNPs, 889,667 indels and 1,327,993 multiallelic sites for the GATK3 VCFs). High quality SNPs and indels were used from the core regions of chromosome 1, excluding hypervariable and subtelomeric regions, after pruning them for linkage disequilibrium and selecting high-quality samples with less than 20% missing genotypes (aiming to reduce potential false noise mostly for indels). The pairwise variance-standardized genetic relationship matrix was calculated and a t-distributed stochastic neighbour embedding (tSNE) analysis was performed using SNPs and indels, individually and in combination. A similar general population structure between sub-Saharan Africa, South-East Asia, South America and Papua New Guinea was observed across all three variant sets (SNPs, indels and SNPs + Indels) as previously reported [6, 20, 21], (Additional file 1: Figure S3). Subsequently, only African samples were selected, as a more challenging population to differentiate, and clustering was performed using tSNE (Fig. 3) and principal component analysis (Additional file 1: Figure S4) to look at continental population structure. SNPs showed more pronounced separation of Democratic Republic of Congo from West African samples (Fig. 3C) whereas indels provided greater differentiation of parasite populations within the same geographical regions (Fig. 3B). Specifically, with indels, the vast majority of samples from Ghana and Guinea were clearly separated from the rest of parasites from West Africa; and Tanzania, Madagascar, Kenya, Ethiopia and Malawi formed more population structure within East and South-East Africa. Interestingly, when combined both SNPs and indels led to higher resolution of population separation within and across the different African regions (Fig. 3A) compared to each of them analysed separately.

### Increasing ploidy improves variant calling performance in unbalanced mixed infections

Given the decreased performances of both GATK4 and GATK3 in mixed infections in diploid mode in general, the pipelines at ploidy 6 to improve detection of low abundance within-sample variants (due to minor low abundance parasites). Since most downstream analytic tools cannot fully process hexaploid VCFs, such as decomposing complex indels into smallest fragments prior to comparing two callsets, the evaluation was limited to SNPs. The total number of additional true positive SNPs detected by the hexaploid mode relative to the diploid one on chromosome 13 was 449, 359, 112, 102, 95 and 78 for 15%:95%, 10%:90%, 15%:85%, 20%:80%, 25%:75% and 50%:50% of IT:KH01 mixed infections, respectively (Additional file 1: Table S3). For the 50%:50% mixed infection sample, the hexaploid mode did not substantially improve variant detection, confirming the hypothesis whereby the diploid mode works more accurately when there are equal proportions of individual strains in the sample. These findings suggest that the polyploid modes would be more suitable for monitoring molecular markers of malaria drug resistance or detection of low abundance strains.

To verify this, the pipeline was applied at ploidy 2 and 6 on 6626 field isolate samples from the MalariaGEN Pf6 release that have been collected from all malaria-endemic regions worldwide [6] to analyse *k13* resistant mutations that have been reported by the WHO [22]. Mutations found by the pipelines were compared to those present in the public GATK3 VCF (diploid mode). As expected, the hexaploid mode of the optimized GATK4 detected 32 additional resistant mutations that were totally missed by GATK3 (Table 1). These resistant samples rescued by the hexaploid mode of the optimized pipeline were confirmed by visualizing their respective BAM files in IGV (examples are illustrated in Additional file 1: Figure S5 for C580Y mutation). Interestingly, one sample from Cameroon in Central West Africa where artemisinin resistance is yet to be reported, classified as wildtype by GATK3, was found with C580Y mutation (Additional file 1: Figure S5).

**Table 1:** Resistant *k13* mutations detected by the optimized GATK4 at hexaploid and diploid modes versus GATK3

K13 mutations	Optimized GATK4 (ploidy6)	Optimized GATK4 (ploidy2)	GATK3
A481V	5	5	5
C580Y	664	649	650
G449A	6	5	5
G538V	24	21	21
I543T	36	34	33
M446I	38	36	36
N458Y	17	17	17
P441L	28	28	28
P553L	24	23	23
P574L	30	30	30
R539T	65	63	62
R561H	24	24	24
Y493H	111	106	106

All the mutations were found in South-East Asia except for one sample carrying the C580Y allele that was collected in Cameroon

### The optimized pipeline with ploidy 6 enables robust estimation of complexity of infections

**Fig. 4:**
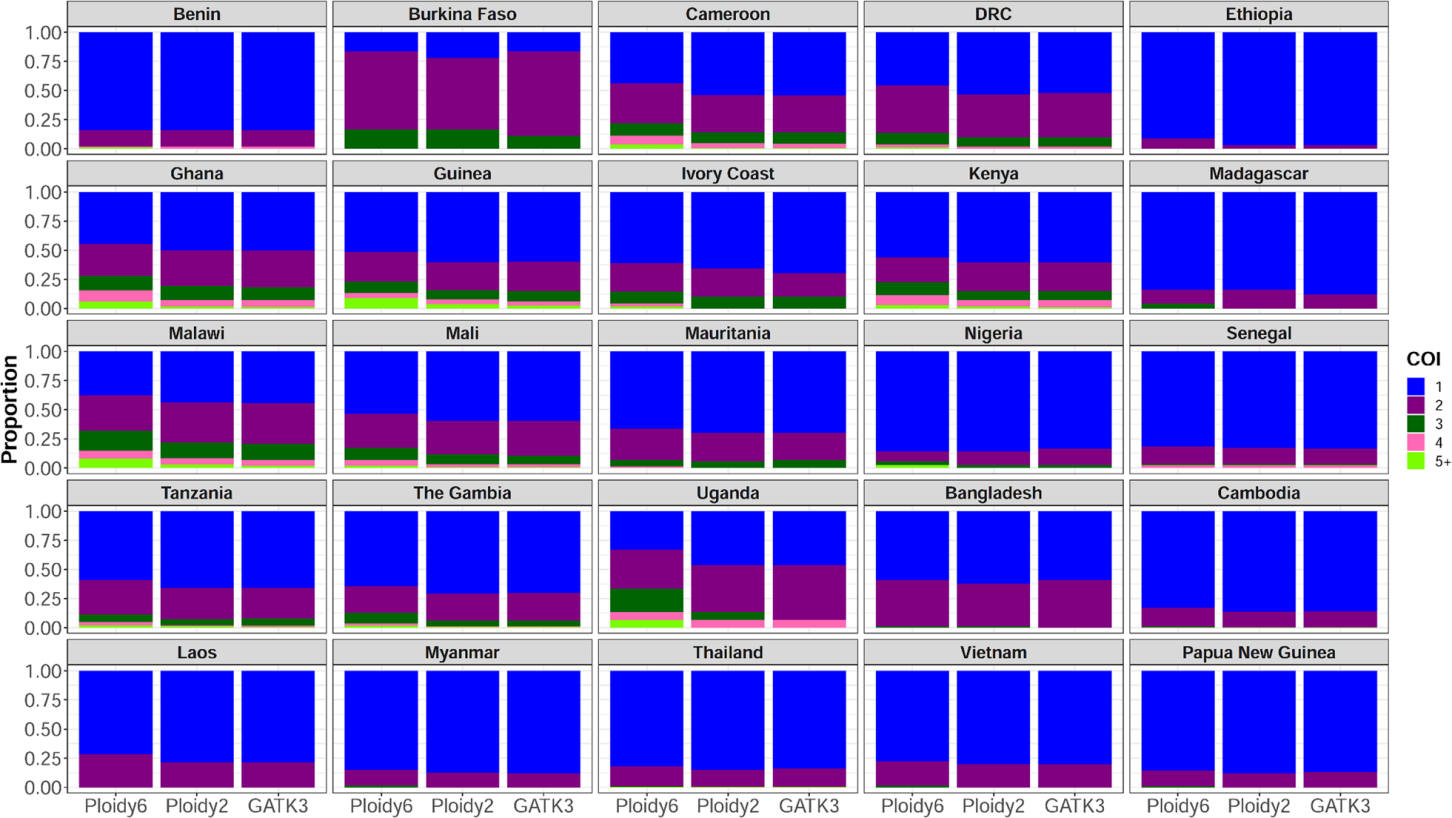
Complexity of infection analysis by site based on the optimized GATK4 pipeline with ploidy of 6 and 2 versus GATK3. This analysis included SNPs with minor allele frequencies > 1% and missingness rates < 10% on the core region of chromosome 13 from public Illumina reads of field isolate samples (n = 6626). Ploidy 6 and ploidy 2 refer to the optimized GATK4 pipeline ran at hexaploid and diploid modes that were compared to GATK3 (publically available callset from MalariaGEN Pf6). Ploidy 6 showed significant increase in polyclonal sample detection compared to ploidy 2 and GATK3 after pairwise statistical analysis between pipelines (P < 0.05, Wilcoxon test). DRC: Democratic Republic of Congo. COI: complexity of infection

To examine the practical benefits of improved variant calling, the overall WGS dataset complexity of infection (COI) was assessed for field isolates from sub-Saharan Africa, South-Asia Asia and South America. COI based was computed based on common SNPs on chromosome 13 called using the optimized pipeline with ploidy of 6 and 2 and the GATK3 at diploid mode. Variants with minor allele frequencies > 1% and missing genotypes > 10% were selected for the COI calculation using the REAL McCOIL package [23]. Overall, more mixed infections were found when the hexaploid callset was used to estimate the COI compared to the other VCFs and these results were consistent across all sampling locations although polyclonality was generally reduced outside sub-Saharan Africa (Fig. 4). The prevalence of monoclonal infections was 52.2%, 58.2% and 58.3% in sub-Saharan African regions using optimized GATK4 with ploidy 6 and ploidy 2 and GATK3, respectively. Regarding biclonal infections in the same regions, 28.2%, 28.6% and 29.2% prevalence was found with the pipeline at ploidy 6 and ploidy 2 and GATK3, respectively. The prevalence of samples with COI > 2 was 19.6%, 13.2% for the hexaploid and diploid modes of the optimized GATK4, respectively, and 12.5% for GATK3. Thus, increasing the ploidy up to 6 significantly increased the detection of polyclonal samples compared to ploidy 2 for GATK4 and GATK3 (P < 0.05, Wilcoxon test) in 8 sampling sites; including Cambodia, Cameroon, Democratic Republic of Congo, Ghana, Guinea, Malawi, Mali and Tanzania.

## Discussion

This study, which aimed to adjust GATK4 settings to *P. falciparum* WGS and develop a new robust training dataset for accurate removal of low-quality variants from raw VCFs, provides an optimized variant calling pipeline that outperforms existing similar resources [6]. Unlike GATK3 and default GATK4, this fully optimized pipeline is equally competent at calling SNPs and indels with high sensitivity. Thus, these results fill an important gap regarding the WGS of *P. falciparum* today and should encourage a wide use of the indels alongside the SNPs to improve the resolution of the genomic epidemiology analyses, especially within closely-related parasite populations. Importantly, the findings show that this optimized pipeline is also more robust with clinical mixed infection samples in which the number and relative frequency of each component strain is variable and undetermined. In further analysis based on field isolate samples, increasing the ploidy values in HaplotypeCaller significantly improves the sensitivity of the variant calls, which might be impactful in tracking drug resistance mutations or estimating COIs, especially in hyperendemic regions in sub-Saharan Africa where artemisinin resistance is actively monitored and mixed infections are common [6, 24].

Although GATK4 with default settings was more sensitive and precise than GATK3 [6] in general, the new training set significantly improved its performance. This achievement can be attributed not only to the sensitivity of GATK4 to keep more likely true variants from mapped reads during the initial HaplotypeCaller and GenotypeVCFs steps but also to the accuracy of the downstream soft filtering of the raw VCFs by the custom training dataset. Interestingly, modifications of parameters that control for base and mapping qualities as well as malaria-adjusted prior heterozygosities improved the sensitivity of the pipeline in longer Illumina reads from both single and mixed infection samples without significant increase in the amount of false variants and thus should work well for all current and future sequencing.

One innovation is a new and simple computational method to synthesize a positive training dataset using accurate PacBio assemblies of laboratory strains as templates that can be reproduced in other organisms in which cross models are unavailable. This errors-depleted benchmark truth callset was able to train the Gaussian mixture model that is implemented in GATK4 to more effectively discriminate between true and false variants. Even though few assemblies were available, the size of the training dataset could be technically increased by adding mixed infection samples simulated from the unique templates with a growing number of available long-read Oxford Nanopore assemblies. Key parameters that were optimized were using a lower variant quality score log-odds (VQSLOD) threshold and increased local assembly size for haplotypecaller to better capture indels. Here 2 kb overlapping artificial reads were generated with 100× coverage but these key parameters can be more flexibly modified based on the goal of the variant calling project, which is hard to obtain with high precision with wet laboratory methods [9, 25, 26].

Given that both SNPs and indels were equally accurate, the impacts of both on population genetics analyses including COI and population structure could be explored confidently. It was found that indels led to more sensitive estimation of complexity of infection, more likely due to the fact that they are more abundant than SNPs in the entire core genome of *P. falciparum* as previously demonstrated [5, 9]. Similarly, SNPs and indels seem to have different impacts on population structure analysis in which the former, which is more stable, provides better separation of populations between different geographic regions but the latter, which occurs more rapidly, produces high resolution of subpopulation detection within the same areas. When combined, it was found that both variants are more beneficial in population structure analysis than using SNP only because effects seem to be additive. Indels seem to show more local inbreeding between parasites on a shorter time-scale that should complement the broader geographic genetic differentiation obtained with SNPs.

As a limitation of all the GATK pipelines (mainly the non-optimized pipelines) tested, the performance of variant calls was relatively lower in mixed than single infection samples. This general issue was partially resolved by increasing the ploidy in the HaplotypeCaller package which also allowed us to detect true artemisinin-resistant SNPs on *k13* gene that were missed by the diploid mode. Additionally, there are currently limited options for the downstream analysis of polyploid VCFs as most of the existing tools were specifically made for the diploid format.

## Conclusions

In conclusion, an optimized variant calling pipeline based on GATK4 that produces high quality SNPs and indel data from monogenomic and mixed infection samples has been created. The output of the pipeline in downstream population genetic analyses using publicly available WGS data demonstrated the value of incorporating indels in such studies alongside SNPs. This pipeline should contribute to improving the quality of *P. falciparum* WGS studies and both the diploid and polyploid methods can be borrowed to analyse the genomic data of other complex microorganisms in which calling accurate variants is elusive.

## Supplementary Information

**Additional file 1: Fig. S1.** Quality of the GATK variant calling on the in silico training dataset as compared to direct calls from assemblies reference call sets within the core genome. **Fig. S2.** Workflow showing different steps in reference truth-set generation for 10 laboratory strains. **Fig. S3.** Malaria parasite population structure of malaria across subcontinents. **Fig. S4.** Visualization of *k13* C580Y mutations detected only by the fully optimized GATK4 pipeline (pipeline 2) at ploidy 6. The mutation consists of the substitution of C by T (red) at position 1,725,259. The visualization was done in IGV. The two samples (PH0949-C and PH1181-C) at the end were detected as mutants by GATK3 but filtered out by the optimized GATK4 due to poor read coverage (2×). **Fig. S5. **Diagram illustrating the fully optimized GATK4 pipeline (pipeline 2). Optimized parameters are highlighted red. **Table S1.** High-quality read data of 10 strains for which there exists accurate PACBIO assemblies. **Table S2.** Quality scores of public shorter illumina read samples. Samples include single (7G8, GB4 and HB3) and mixed infections of 7G8 and HB3 at different proportions. **Table S3.** Performance of SNP calling in simulated mixed infection samples from IT and KH01 laboratory strains using the optimized pipeline2 at ploidy 6 and 2.

## Data Availability

Data and codes are available at: https://github.com/Karaniare/Optimized_GATK4_pipeline.

## Abbreviations

AT: Adenine–Thymine
COI: Complexity of infection
DRC: Democratic Republic of Congo
GATK: Genomic analysis toolkit
Indel: Insertion–deletion
K13: Kelch 13
MalariaGEN: Malaria Genomic Epidemiology Network
POS: Position
REF: Reference
SNP: Single nucleotide polymorphism
tSNE: T-Distributed stochastic neighbour embedding
VCF: Variant call format
VQSLOD: Variant quality score log-odds
WGS: Whole genome sequencing

## References

[1] WHO. World malaria report 2021. Geneva: World Health Organization; 2021. https://apps.who.int/iris/bitstream/handle/10665/350147/9789240040496-eng.pdf.

[2] Gamboa D, Ho M-F, Bendezu J, Torres K, Chiodini PL, Barnwell JW (2010). A large proportion of *P. falciparum* isolates in the Amazon region of Peru lack pfhrp2 and pfhrp3: implications for malaria rapid diagnostic tests. PLoS ONE.

[3] Koita OA, Doumbo OK, Ouattara A, Tall LK, Konaré A, Diakité M (2012). False-negative rapid diagnostic tests for malaria and deletion of the histidine-rich repeat region of the hrp2 gene. Am J Trop Med Hyg.

[4] Dondorp AM, Nosten F, Yi P, Das D, Phyo AP, Tarning J (2009). Artemisinin resistance in *Plasmodium falciparum* malaria. N Engl J Med.

[5] Gardner MJ, Hall N, Fung E, White O, Berriman M, Hyman RW (2002). Genome sequence of the human malaria parasite *Plasmodium falciparum*. Nature.

[6] Ahouidi A, Ali M, Almagro-Garcia J, Amambua-Ngwa A, Amaratunga C, MalariaGEN (2021). An open dataset of *Plasmodium falciparum* genome variation in 7,000 worldwide samples. Wellcome Open Res.

[7] Ariey F, Witkowski B, Amaratunga C, Beghain J, Langlois A-C, Khim N (2014). A molecular marker of artemisinin-resistant *Plasmodium falciparum* malaria. Nature.

[8] Amambua-Ngwa A, Amenga-Etego L, Kamau E, Amato R, Ghansah A, Golassa L (2019). Major subpopulations of *Plasmodium falciparum* in sub-Saharan Africa. Science.

[9] Miles A, Iqbal Z, Vauterin P, Pearson R, Campino S, Theron M (2016). Indels, structural variation, and recombination drive genomic diversity in *Plasmodium falciparum*. Genome Res.

[10] Hamilton WL, Claessens A, Otto TD, Kekre M, Fairhurst RM, Rayner JC (2017). Extreme mutation bias and high AT content in *Plasmodium falciparum*. Nucleic Acids Res.

[11] DePristo MA, Zilversmit MM, Hartl DL (2006). On the abundance, amino acid composition, and evolutionary dynamics of low-complexity regions in proteins. Gene.

[12] Felger I, Smith T, Edoh D, Kitua A, Alonso P, Tanner M (1999). Multiple *Plasmodium falciparum* infections in Tanzanian infants. Trans R Soc Trop Med Hyg.

[13] Van der Auwera GA, O’Connor BD (2020). Genomics in the cloud: using docker, GATK, and WDL in Terra.

[14] Poplin R, Ruano-Rubio V, DePristo MA, Fennell TJ, Carneiro MO, Van der Auwera GA, et al. Scaling accurate genetic variant discovery to tens of thousands of samples. bioRxiv. 2018. p. 201178. https://www.biorxiv.org/content/10.1101/201178v3. Accessed 21 Apr 2022.

[15] Otto TD, Böhme U, Sanders M, Reid A, Bruske EI, Duffy CW (2018). Long read assemblies of geographically dispersed *Plasmodium falciparum* isolates reveal highly structured subtelomeres. Wellcome Open Res.

[16] Vembar SS, Seetin M, Lambert C, Nattestad M, Schatz MC, Baybayan P (2016). Complete telomere-to-telomere de novo assembly of the *Plasmodium falciparum* genome through long-read (>11 kb), single molecule, real-time sequencing. DNA Res.

[17] Walliker D, Quakyi IA, Wellems TE, McCutchan TF, Szarfman A, London WT (1987). Genetic analysis of the human malaria parasite *Plasmodium falciparum*. Science.

[18] Wellems TE, Panton LJ, Gluzman IY, do Rosario VE, Gwadz RW, Walker-Jonah A (1990). Chloroquine resistance not linked to mdr-like genes in a *Plasmodium falciparum* cross. Nature.

[19] Hayton K, Gaur D, Liu A, Takahashi J, Henschen B, Singh S (2008). Erythrocyte binding protein PfRH5 polymorphisms determine species-specific pathways of *Plasmodium falciparum* invasion. Cell Host Microbe.

[20] Manske M, Miotto O, Campino S, Auburn S, Almagro-Garcia J, Maslen G (2013). Analysis of *Plasmodium falciparum* diversity in natural infections by deep sequencing. Nature.

[21] Miotto O, Amato R, Ashley EA, MacInnis B, Almagro-Garcia J, Amaratunga C (2015). Genetic architecture of artemisinin-resistant *Plasmodium falciparum*. Nat Genet.

[22] WHO (2020). Report on antimalarial drug efficacy, resistance and response: 10 years of surveillance (2010–2019).

[23] Chang H-H, Worby CJ, Yeka A, Nankabirwa J, Kamya MR, Staedke SG (2017). THE REAL McCOIL: a method for the concurrent estimation of the complexity of infection and SNP allele frequency for malaria parasites. PLoS Comput Biol.

[24] Mobegi VA, Loua KM, Ahouidi AD, Satoguina J, Nwakanma DC, Amambua-Ngwa A (2012). Population genetic structure of *Plasmodium falciparum* across a region of diverse endemicity in West Africa. Malar J.

[25] Zook JM, Salit M (2011). Genomes in a bottle: creating standard reference materials for genomic variation—why, what and how?. Genome Biol.

[26] Li H, Bloom JM, Farjoun Y, Fleharty M, Gauthier L, Neale B (2018). A synthetic-diploid benchmark for accurate variant-calling evaluation. Nat Methods.

