# Supplementary material for: An optimized GATK4 pipeline for Plasmodium falciparum whole genome sequencing variant calling and analysis
Source: Malar J. 2023 Jul 7;22:207. doi: 10.1186/s12936-023-04632-0 (PMC10327343; doi:10.1186/s12936-023-04632-0)
Supplement: Supplementary file 1 — Additional file 1: Fig. S1. Quality of the GATK variant calling on the in silico training dataset as compared to direct calls from assemblies reference call sets within the core genome. Fig. S2. Workflow showing different steps in reference truth-set generation for 10 laboratory strains. Fig. S3. Malaria parasite population structure of malaria across subcontinents. Fig. S4. Visualization of k13 C580Y mutations detected only by the fully optimized GATK4 pipeline (pipeline 2) at ploidy 6. The mutation consists of the substitution of C by T (red) at position 1,725,259. The visualization was done in IGV. The two samples (PH0949-C and PH1181-C) at the end were detected as mutants by GATK3 but filtered out by the optimized GATK4 due to poor read coverage (2×). Fig. S5. Diagram illustrating the fully optimized GATK4 pipeline (pipeline 2). Optimized parameters are highlighted red. Table S1. High-quality read data of 10 strains for which there exists accurate PACBIO assemblies. Table S2. Quality scores of public shorter illumina read samples. Samples include single (7G8, GB4 and HB3) and mixed infections of 7G8 and HB3 at different proportions. Table S3. Performance of SNP calling in simulated mixed infection samples from IT and KH01 laboratory strains using the optimized pipeline2 at ploidy 6 and 2. [file 12936_2023_4632_MOESM1_ESM.pdf]

An Optimized GATK4 Pipeline for *Plasmodium falciparum* Whole Genome Sequencing Variant Calling and Analysis.

K. Niaré et al.

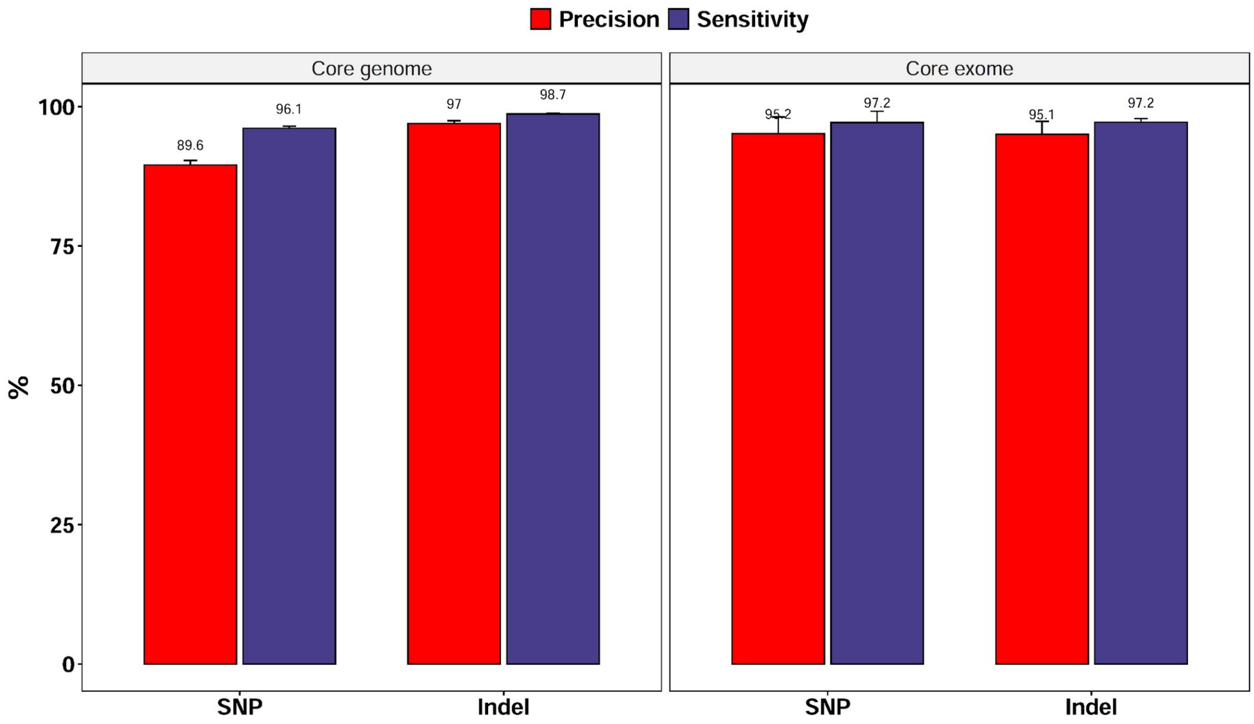

Fig. S 1: Quality of the GATK variant calling on the in silico training dataset as compared to direct calls from assemblies reference call sets within the core genome. Core genome excludes subtelomeric and internal hypervariable regions. Core exome corresponds to all coding sequences within the core genome. PACBIO assemblies for 10 laboratory strains (7G8, Dd2, GA01, GB4,GN01, HB3, IT, KH01, KH02 and SN01) were included in the analysis. For each strain, *in silico* synthetic 2kb overlapping reads at 100X coverage to the genome were used to call variants with GATK version 4.

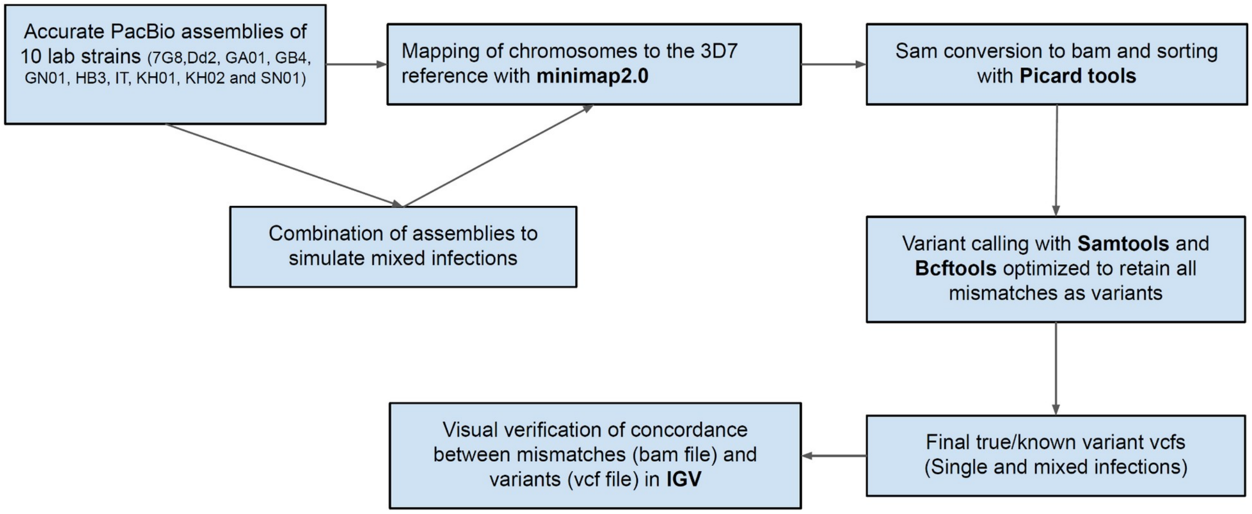

Fig. S2: Workflow different steps in reference thruth set for 10 laboratory strains.

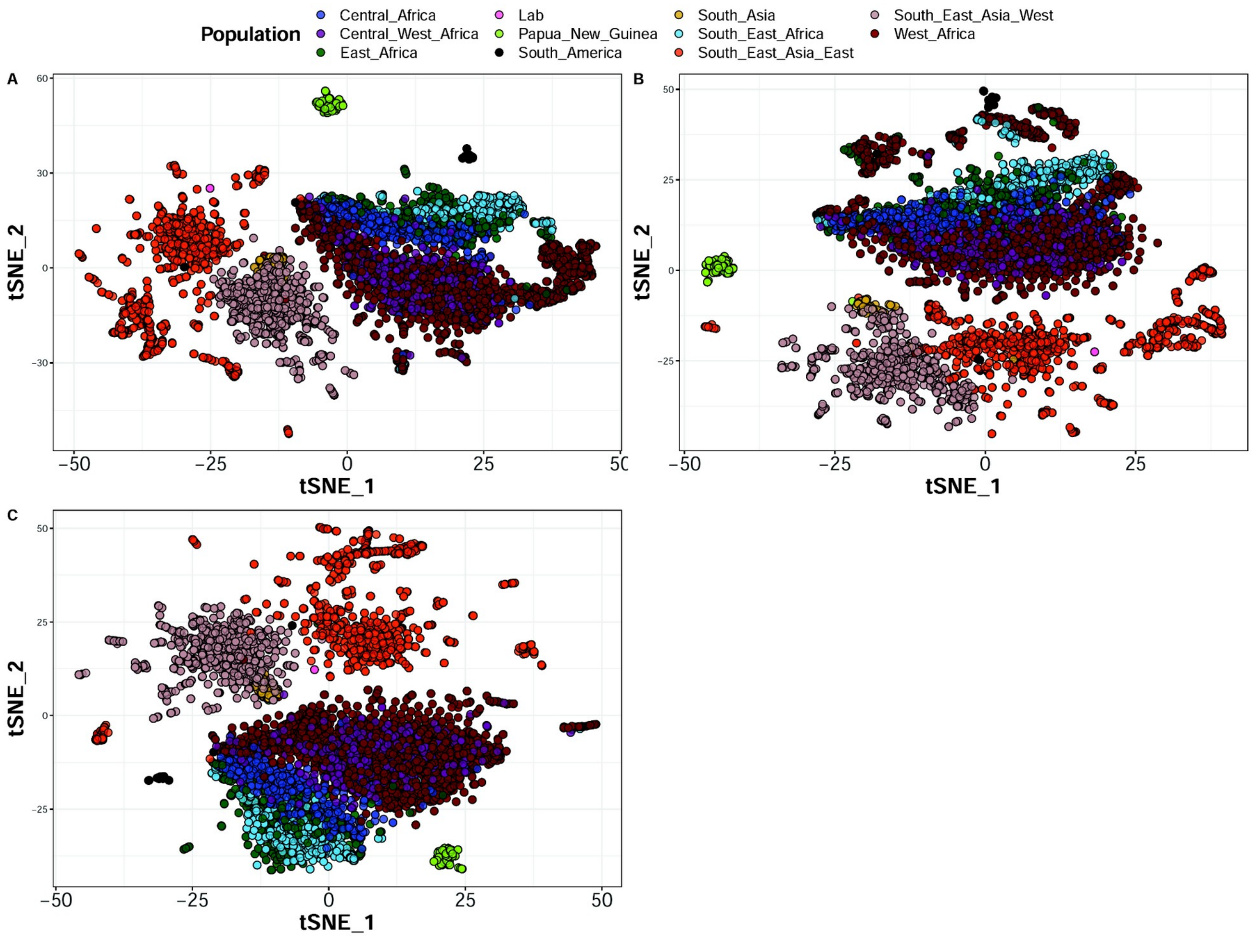

Fig. S3: Malaria parasite population structure of malaria across subcontinents. t-distributed stochastic neighbor embedding (tSNE) was computed from the variance-standardized genetic relationship matrix generated using **A)** SNPs and indels combined, **B)** indels only and **C)** SNPs only. Variant data from chromosome 1 were pruned for linkage disequilibrium and only samples with less than 20% missing genotypes were kept.

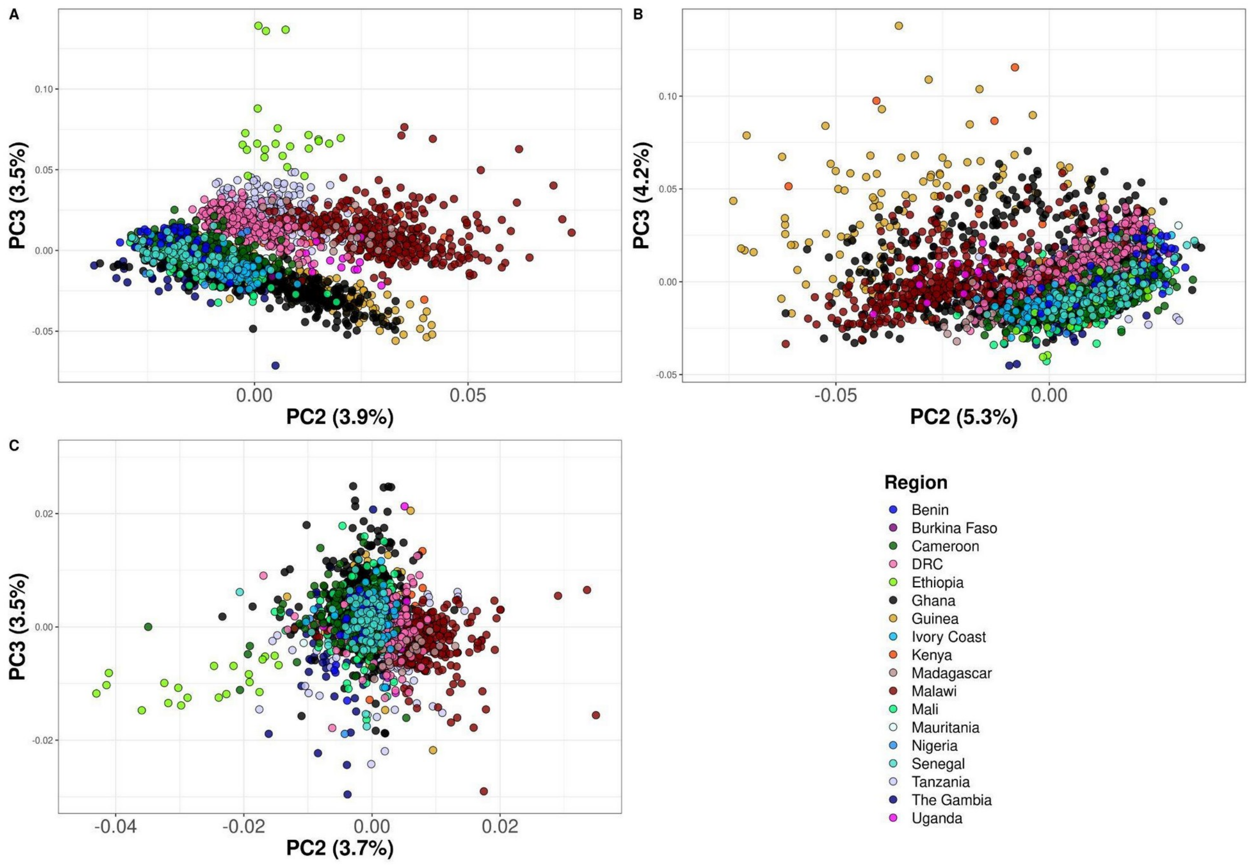

Fig. S4: Principal component analysis showing local population structure within sub-Saharan Africa. A) SNPs and indels combined, **B)** indels only and **C)** SNPs only. Variant data from chromosome 1 were pruned for linkage disequilibrium and only samples with less than 20% missing genotypes were kept. DRC: Democratic Republic of Congo.

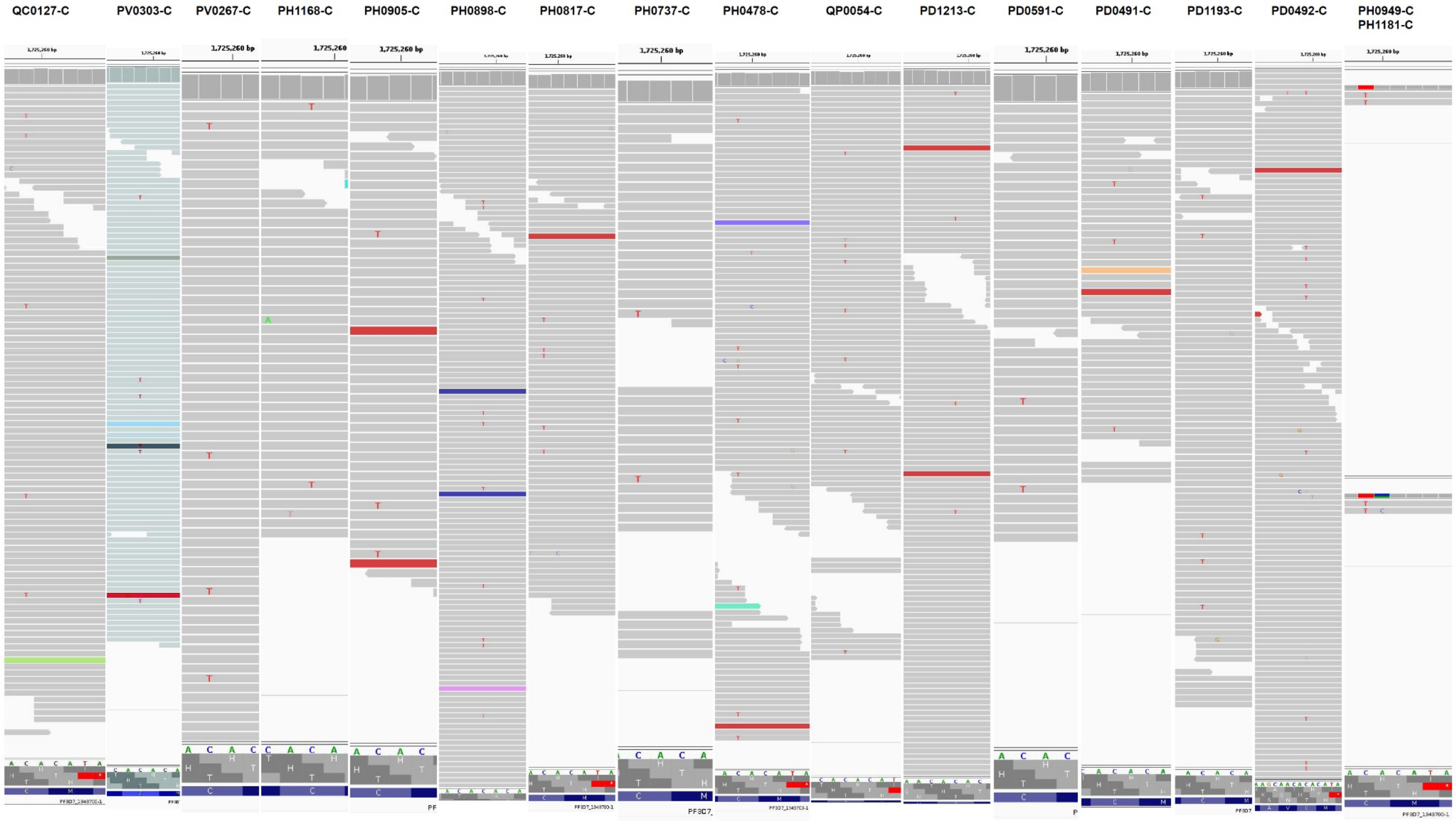

Fig. S4: Visualization of *k13* C580Y mutations detected only by the fully optimized GATK4 pipeline (pipeline 2) at ploidy 6. The mutation consists of the substitution of C by T (red) at position 1,725,259. The visualization was done in IGV. The two samples (PH0949-C and PH1181-C) at the end were detected as mutants by GATK3 but filtered out by the optimized GATK4 due to poor read coverage (2X)

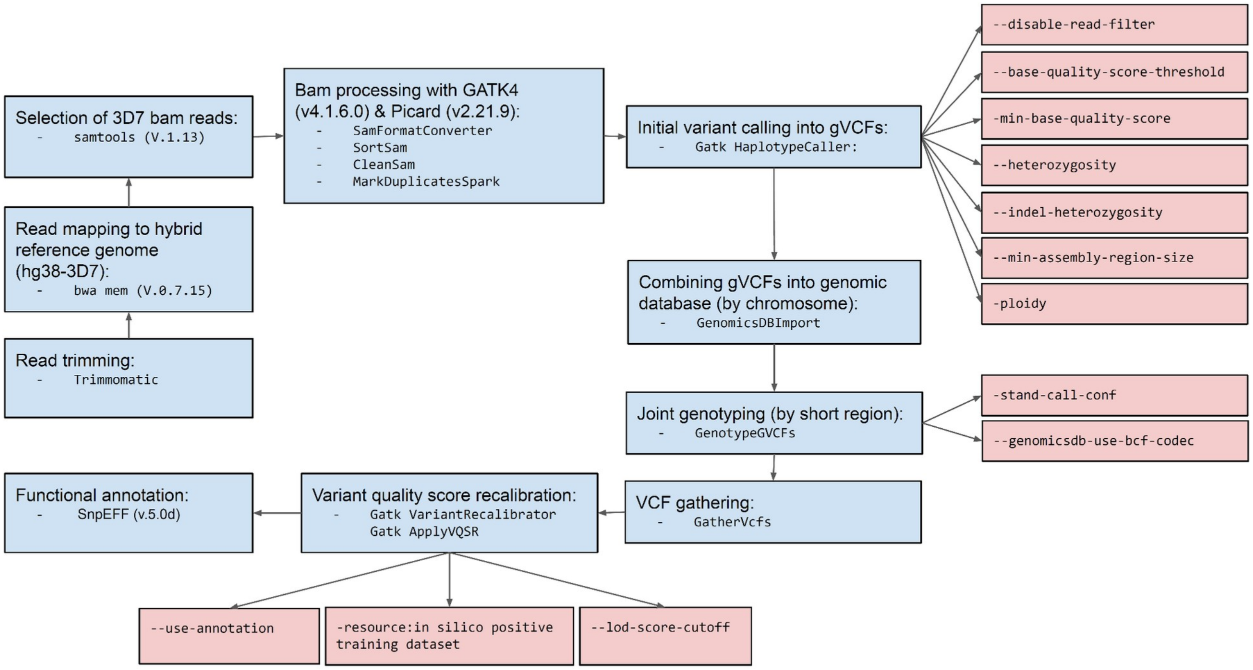

Fig. S5: Diagram illustrating the fully optimized GATK4 pipeline (pipeline 2). Optimized parameters are highlighted red.

Supplementary tables

Table S1: High-quality read data of 10 strains for which there exists accurate PACBIO assemblies.

Strains	Read Coverage First quartile	Read coverage Median	Read coverage Third quartile	Insert size Median	Insert size Median absolute deviation	Read length Mean	Read quality Mean
**7G8**	48	56	64	524	108	250	33.8
**Dd2**	44	52	59	505	99	250	34
**GA01**	38	44	50	504	99	250	33.9
**GB4**	36	44	50	524	104	250	33.4
**GN01**	29	35	40	505	87	250	33.6
**HB3**	30	36	42	489	94	250	33.6
**IT**	159	181	199	405	83	250	33.9
**KH01**	63	81	100	435	113	250	34.2
**KH02**	31	37	43	464	82	250	33.9
**SN01**	43	51	58	460	76	250	34.3

Table S2: Quality scores of public shorter illumina read samples. Samples include single (7G8, GB4 and HB3) and mixed infections of 7G8 and HB3 at different proportions.

Strains	Read Coverage First quartile	Read coverage Median	Read coverage Third quartile	Insert size Median	Insert size Median absolute deviation	Read length Mean	Read quality Mean
**7G8**	61	104	137	230	37	100	34.2
**GB4**	60	101	131	314	36	76	34.3
**HB3**	68	129	173	224	37	100	34.2
**90%HB3+10%7G8**	71	133	174	229	37	100	34.2
**50%HB3+50%7G8**	71	111	139	227	37	100	34.2
**25%HB3+75%7G8**	69	112	144	224	37	100	34.2
**5%HB3+95%7G8**	60	116	162	230	37	100	34.2

Table S3: Performance of SNP calling in simulated mixed infection samples from IT and KH01 laboratory strains using the optimized pipeline2 at ploidy 6 and 2

**Mixed infections**	**True Positives**		**False positive**		**Variants rescued with ploidy 6**	**Extra false positives with ploidy 6**
	**Ploidy 6**	**Ploidy 2**	**Ploidy 6**	**Ploidy 2**		
**5%IT:95%KH01**	2646	2197	509	161	449	348
**10%IT:90%KH01**	2859	2500	894	231	359	663
**15%IT:85%KH01**	2848	2736	570	344	112	226
**20%IT:80%KH01**	2890	2788	1322	367	102	955
**25%IT:75%KH01**	2913	2818	852	369	95	483
**50%IT:50%KH01**	2912	2834	721	371	78	350
